# Gambling disorder in a university hospital setting: a retrospective analysis of patient characteristics

**DOI:** 10.3389/fpsyt.2025.1723070

**Published:** 2026-02-05

**Authors:** Damla Isman Haznedaroglu, Okan Gorkem, Ozgur Degirmenci

**Affiliations:** Department of Psychiatry, Ege University, Izmir, Türkiye

**Keywords:** gambling disorder (GD), gambling disorder; comorbidity, gambling disorder; suicidality, pathological gambling, retrospective study

## Abstract

**Background:**

Gambling Disorder (GD), classified within the behavioral addiction spectrum, has gained increasing clinical attention; however, studies examining its psychiatric comorbidities and sociodemographic correlates in Türkiye remain limited.

**Methods:**

This study aimed to investigate the psychiatric comorbidities, substance use patterns, and sociodemographic characteristics of patients diagnosed with GD. This retrospective cross-sectional chart review was conducted at the Addiction Clinic, Department of Psychiatry, Ege University Faculty of Medicine, Türkiye. Ninety-five patients aged 18–65 years who met the Diagnostic and Statistical Manual of Mental Disorders, Fifth Edition (DSM-5) criteria for GD were included, while those whose gambling behavior was influenced by medical or psychiatric episodes (e.g., mania, psychosis) or cognitive impairment were excluded. Sociodemographic data, comorbid psychiatric diagnoses, alcohol and substance use, suicide attempts, and family history of addiction were evaluated using logistic regression and chi-square analyses.

**Results:**

The sample was predominantly male (96.8%) with a mean age of 35 years. Middle school graduates had a 6.9-fold higher risk of Alcohol Use Disorder (AUD) than university graduates (odds ratio [OR]=6.875, p=0.017). Cannabis use was significantly associated with alcohol consumption at both social drinking and AUD levels (p<0.001). Suicide attempts were nearly three times more likely among patients with depression (OR=2.949, p=0.048).

**Conclusions:**

Patients with GD exhibited high rates of comorbid alcohol and substance use disorders. Lower educational attainment and depression were associated with greater risk, while the cannabis–alcohol association underscored the interconnectedness of addictive behaviors. These findings highlight the importance of comprehensive biopsychosocial assessment and prospective studies to guide prevention and individualized treatment strategies.

## Introduction

Gambling Disorder (GD), according to the DSM-5, is defined as a pattern of persistent and recurrent gambling behavior leading to clinically significant distress or impairment (1). For a diagnosis to be established, at least four of the nine criteria outlined in the DSM-5 must be met within a 12-month period. The disorder can present episodically or continuously and is classified as mild, moderate, or severe depending on the number of criteria endorsed. With the advent of DSM-5, the term previously known as “pathological gambling” in DSM-III, DSM-IV, and ICD-10 has been reclassified as “gambling disorder” and moved from the category of impulse control disorders to the group of substance-related and addictive disorders (2). This reclassification reflects the longstanding conceptualization of GD as an addictive disorder. Indeed, GD shares several commonalities with alcohol and substance use disorders (AUD/SUD), including high comorbidity rates, overlapping diagnostic criteria, shared genetic vulnerabilities, similar neurobiological mechanisms, and partially convergent treatment approaches (2, 3).

In addition, the literature also describes the concept of “problem gambling” for individuals who do not meet the full diagnostic threshold yet experience gambling-related difficulties (2). Even subthreshold gambling behaviors may lead to significant adverse health outcomes (2, 4), paralleling the harms observed in mild substance use disorders. One contributing factor is that the DSM-5 requires a higher symptom threshold for GD (≥4 criteria) compared to substance use disorders.

A meta-analysis of studies published between 2016 and 2022 found the prevalence of moderate-risk gambling in the adult population to be 2.43%, while the prevalence of problem/pathological gambling was 1.29% (5). In a Turkish sample of 19,825 individuals, the prevalence of problem gambling was reported as 19% (6). The highest risk of behavioral addiction was observed among male high school graduates (15.7%), whereas the lowest risk was reported among women with primary school or lower education (92.1% with no symptoms). Another nationwide representative survey in Türkiye found that 6.8% of participants had gambled at least once in their lifetime, and 2% continued gambling regularly (7). Furthermore, individuals who had gambled exhibited significantly higher rates of tobacco, alcohol, and substance use, as well as trauma and psychiatric history, compared to those who had never gambled.

Over the last three decades, a notable increase in gambling behavior has been observed, with gambling initiation occurring at younger ages (8). The rise of online gambling has contributed to the emergence of novel, higher-risk forms of addiction (9).

Psychiatric comorbidity rates are markedly high among individuals diagnosed with pathological gambling; approximately 96% have at least one, and 64% present with three or more psychiatric disorders (2). Substance use disorders, impulse control disorders, mood disorders, and anxiety disorders are particularly prevalent.

Today, gambling has become a major public health concern, causing significant financial, relational, psychological, and legal harms to individuals and society (10). Although early identification and intervention are crucial, public awareness regarding the symptoms and consequences of gambling remains inadequate. Understanding the individual and environmental factors influencing gambling behavior is therefore essential for prevention. Current evidence indicates that in Türkiye, not only the overall prevalence but also the characteristics of subgroups of individuals with GD warrant detailed examination.

Research investigating psychiatric comorbidities and sociodemographic characteristics of patients with GD in Türkiye remains limited, creating a significant knowledge gap. This study aims to contribute to the literature by examining psychiatric comorbidities and sociodemographic features of individuals diagnosed with GD in Türkiye.

## Methods

The study included patients aged 18–65 who were followed up at the Addiction Clinic of Ege University Faculty of Medicine, Department of Psychiatry, and diagnosed with GD according to DSM-5 criteria. Individuals exhibiting gambling escalation during manic, depressive, or psychotic episodes, those with gambling behaviors influenced by medical conditions, or those with cognitive impairments due to illness, surgery, or trauma were excluded. Retrospective examination of outpatient records, laboratory results, and consultation notes was conducted. Sociodemographic data, DSM-5 comorbid diagnoses, suicide attempt history, alcohol and substance use information, and family history of addiction were assessed. Alcohol use was coded from clinician documentation in the clinical records as no alcohol use, social drinker, or alcohol use disorder. The category “social drinker” was assigned when alcohol use was described as social/occasional and mild-moderate (up to two standard drinks per day for men and one for women), with no chart evidence of binge drinking episodes or alcohol-related behavioral impairment. For analyses requiring a binary variable, alcohol use was also examined as alcohol use disorder (present vs absent). Indebtedness was extracted from clinical records as a binary variable (yes/no) using the chart item indicating the presence of debt. The study protocol was approved by the Ethics Committee for Medical Research of Ege University Faculty of Medicine (Decision No: 2025-4692) and conducted in accordance with the Declaration of Helsinki. This retrospective study was initiated after obtaining ethical approval in 2025 and covered patient records from January 1, 2020, to December 31, 2024. Informed consent was obtained from all participants after ethical approval, during routine follow-up visits at our clinic, and prior to inclusion of their anonymized data in the study. This study was not pre-registered, and the analyses should be considered exploratory.

## Results

The sociodemographic characteristics of the participants are summarized in **Table 1**. A total of 95 patients were included, of whom 96.8% were male (n = 92) and 3.2% female (n = 3). Nearly half were married (49.5%), 40% single, and 10.5% divorced. Regarding education, 44.2% were high school graduates, 38.9% university graduates, 11.6% middle school graduates, and 5.3% primary school graduates. The majority were employed (74.7%), 20% unemployed, and 5.3% retired.

**Table 1:** Sociodemographic characteristics.

Variables		n	%	Mean age ± SD (Min-Max)
Gender	Male	92	96.8	35 ± 10 (18–62)
	Female	3	3.2	32 ± 6 (26–38)
Marital Status	Single	38	40	28 ± 6 (18–43)
	Married	47	49.5	40 ± 9 (23–62)
	Divorced	10	10.5	40 ± 8 (24–55)
Educational Level	Primary school graduate	5	5.3	46 ± 14 (28–62)
	Secondary school graduate	11	11.6	33 ± 7 (20-42)
	High school graduate	42	44.2	34 ± 9 (18-55)
	University graduate	37	38.9	35 ± 10 (22-60)
Employment Status	Employed	71	74.7	35 ± 8 (18-55)
	Unemployed	19	20	31 ± 13 (19-62)
	Retired	5	5.3	49 ± 12 (29-60)

SD, Standard Deviation.

A statistically significant association was found between educational level and AUD (p = 0.037). Logistic regression showed borderline significance in predicting AUD (Omnibus χ² (3) = 7.15, p = 0.065), explaining 11.7% of the variance. Middle school graduates had a 6.9-fold higher risk of developing AUD compared to university graduates (OR = 6.875, 95% CI [1.421–33.261], p = 0.017). Primary school graduates also showed an elevated risk (OR = 5.500, 95% CI [0.695–43.514]), though not statistically significant (p = 0.106). No significant difference was observed between high school and university graduates (OR = 1.650, 95% CI [0.442–6.160], p = 0.456).

Among those with a family history of substance use, the prevalence of SUD was 75%, compared to 19.8% among those without such a history (Fisher’s Exact Test, p = 0.033). Logistic regression showed a statistically significant association between family history and SUD (Nagelkerke R² = 0.084; B = -2.499, p = 0.035; OR = 0.082, 95% CI [0.008–0.837]). However, the small number of participants with a family history (n = 4) may have influenced the stability and direction of the regression coefficients, as indicated by the wide confidence interval.

The association between alcohol consumption and family history of alcohol use varied by categorization. Family history of alcohol use was recorded dichotomously as the presence or absence of AUD. In contrast, patients’ alcohol use was categorized in two ways: first as a binary variable (AUD present or absent) and then using a three-level classification (none, social drinker, or AUD). When alcohol use was analyzed only in terms of AUD diagnosis, there was no significant association with family history (p = 0.394). However, when analyzed in the three-level format, the association reached significance (p = 0.027). This suggests that individuals with a family history of AUD may generally engage more frequently in alcohol consumption behaviors.

A significant association was observed between SUD and AUD (Fisher’s Exact Test, p = 0.003). Logistic regression showed that while SUD predicted AUD, the model had limited explanatory power (14.2%). Results indicated an inverse relationship, with SUD being associated with a lower likelihood of AUD (B = -1.689, p = 0.003; OR = 0.185, 95% CI [0.061–0.560]).

Cannabis use was significantly associated with alcohol consumption in both binary (AUD yes/no) and three-category (none/social drinker/AUD) models. In the binary model, cannabis users had a higher rate of AUD diagnosis (χ² (1) = 7.311, OR = 4.333, 95% CI [1.421–13.217], p = 0.007). In the three-category model, differences were also significant (χ² (2) = 15.396, p < 0.001). Among cannabis users, 40% had AUD, compared to 13.3% among non-users. Multinomial logistic regression revealed that cannabis users were 9.8 times more likely to be social drinkers (OR = 0.102, 95% CI [0.021–0.508], p = 0.005) and 17.2 times more likely to develop AUD (OR = 0.058, 95% CI [0.011–0.317], p = 0.001). The explanatory power of the model was 18.7%.

In total, 17 out of 95 patients (17.9%) had a history of suicide attempt. As all participants were diagnosed with Gambling Disorder, comparisons with non-GD individuals were not applicable. Suicide attempts were marginally associated with depression (χ² (1) = 3.693, p = 0.055; Fisher’s Exact Test, p = 0.048). Individuals with a suicide attempt were nearly three times more likely to have a depression diagnosis (OR = 2.949, 95% CI [0.948–9.171]). The wide confidence interval suggests limited statistical power. Suicide attempts were not associated with indebtedness, alcohol use, or substance use.

No significant associations were found between alcohol/substance use or suicide attempts and employment or marital status. Similarly, no significant differences were detected between comorbid psychiatric diagnoses and measured variables.

## Discussion

The relationship between GD and other addictive disorders has long been recognized, with evidence suggesting shared neurobiological underpinnings (2, 4). Our findings that cannabis use is strongly associated with alcohol consumption—from social drinking to AUD—further support this overlap. Prior literature has also consistently highlighted the robust relationship between alcohol and cannabis use (11).Moreover, alcohol, cannabis, and nicotine are reported as the most common comorbid addictions in GD (12). Our results underscore that alcohol use, even at the social drinking level, is significantly linked with cannabis use, highlighting the clinical relevance of their co-occurrence in GD.

The inverse association between SUD and AUD should be interpreted cautiously. The regression model had limited explanatory power, and the modest sample size—together with small cell counts for substances other than cannabis—may have affected the stability of the estimate. In our sample, cannabis use showed a positive relationship with alcohol use, whereas this pattern was not observed when SUD was examined as an aggregated category; heterogeneity within the SUD group and sparse data for specific substances may therefore have contributed to the counterintuitive inverse association. Future studies with larger samples and substance-specific, structured assessments are needed to clarify whether this finding persists.

A family history of alcohol or substance use was associated with significantly elevated risk of SUD among GD patients (75% vs. 19.8%). Previous studies have suggested that familial influences operate not only through genetic mechanisms but also via modeling, altered normative perceptions, and increased tolerance toward risky behaviors (13). Childhood perceptions of parental gambling, alcohol, and substance use behaviors have also been shown to predict gambling frequency and problem severity in adulthood (14). Thus, witnessing addictive behaviors within the family may elevate risk not only for the same but also for cross-addictive behaviors. Our findings align with this literature and suggest that family history may affect not only substance use but also alcohol consumption behaviors in individuals with GD, reinforcing the possibility of a shared neurobiological substrate.

Genetic predisposition to GD is largely associated with broader behavioral tendencies such as impulsivity and negative affectivity (4). The Taq A1 allele of the dopamine D2 receptor gene, for instance, has been implicated in both GD and AUD, and carriers have been shown to exhibit heightened impulsivity (15). These observations suggest that addictive behaviors are shaped not only by environmental and psychological influences but also by shared neurobiological mechanisms. The three principal circuits underlying addiction—the basal ganglia, extended amygdala, and prefrontal cortex—respectively regulate reward, negative affect, and impulse control, and exhibit functional dysregulation in both substance use and behavioral addictions such as pathological gambling (16). Large-scale studies confirm these findings, showing that approximately 45% of GD patients have a history of AUD or SUD, representing a 2.34-fold higher prevalence compared to controls (17).

Contrary to earlier reports that first-degree relatives of GD patients exhibit elevated rates of GD (2, 4, 14), our data did not yield significant results in this regard. This discrepancy may stem from factors such as sample size, clinical recruitment profiles, or regional differences. Nonetheless, our findings emphasize the need to carefully assess substance use history in GD patients despite this limitation.

Educational level emerged as a significant factor associated with AUD in GD. Middle school graduates were found to be nearly seven times more likely to develop AUD than university graduates, while no significant differences were observed between high school and university graduates. These findings support the role of low education as a risk factor for AUD in GD, potentially due to greater vulnerability to risky behaviors and limited coping strategies. However, given that our sample comprised only GD patients, we cannot directly infer the impact of education on gambling initiation itself. In line with this, Kessler et al. (2008) reported that individuals who were neither university graduates nor students had significantly higher risk of pathological gambling in a U.S. representative sample (18). Similarly, Sert et al. (2024) found that Turkish university students with academic delays were more vulnerable to problem gambling (19). These results suggest that academic difficulties, lack of motivation, and uncertainty about the future may predispose individuals to risky behaviors. Still, as most of our sample had at least high school education, this may reflect that clinical referrals largely comprise severe cases of GD. Population-based studies are needed to clarify the role of education in gambling onset and progression.

Suicide attempts and depression are known to be highly prevalent in GD (2, 12). A systematic review on gambling-related suicidality identified indebtedness and resultant hopelessness as the most frequently cited factors (20). Interestingly, the review also noted that elevated suicide rates occur even among GD patients without depression, suggesting that the depression–suicide link may not be as robust as often presumed. Our results, showing only marginal significance in this association, align with this interpretation. Moreover, we did not observe a significant association between indebtedness and suicide attempts. This may reflect limitations of the retrospective design, small sample size, and binary measurement of indebtedness.

The classification of GD and other behavioral addictions is increasingly grounded in neurobiological and genetic evidence. This underscores the need for integrated biopsychosocial approaches across the addiction spectrum. Improved understanding of genetic predispositions will facilitate the development of personalized treatment strategies.

Our findings highlight the high prevalence and clinical relevance of comorbid alcohol and substance use disorders among GD patients, supporting the inclusion of routine alcohol and substance screening procedures in clinical practice. Joint assessment of psychiatric comorbidities and sociodemographic variables may contribute to the development of more predictive models in addiction research. In addition, recognizing these clinical patterns may help clinicians identify patients at higher risk for depression or suicidality, guide early intervention, and inform individualized treatment planning. Future studies should aim to develop structured assessment tools and integrated treatment approaches that address both gambling behavior and its psychiatric correlates.

## Limitations

The limitations of this study include its modest sample size, restriction to treatment-seeking individuals from a single addiction clinic, and lack of population-based data. Additionally, the overwhelmingly male sample should be highlighted more explicitly as a limitation, particularly regarding the generalizability of findings to female patients with GD. The cross-sectional design also precludes causal inferences. The lower-than-expected prevalence of family history may reflect recall bias, cultural norms, or reluctance to disclose stigmatized information. Furthermore, the absence of a non-gambling comparison group and the binary measurement of indebtedness represent additional limitations, highlighting the need for future studies with broader sampling and more detailed assessments of gambling-related harm and suicidality.

## Conclusion

Overall, these findings emphasize the importance of assessing GD patients not only in terms of gambling behaviors but also with respect to alcohol and substance use, mood disorders, educational background, and genetic vulnerabilities. Such a holistic perspective may strengthen not only treatment efforts but also early risk identification and preventive interventions. Prospective, longitudinal, and population-based studies will be essential to identify risk factors and develop preventive strategies. Such research will enhance understanding of the dynamic nature of behavioral addictions and support the design of individualized treatment plans.

## Data Availability

The raw data supporting the conclusions of this article will be made available by the authors, without undue reservation.
